# How well do participants understand the questions asked in the Online Personal Utility Functions (OPUF) approach? A cognitive debrief of the EQ-HWB-S (EQ Health and Wellbeing Short version) valuation

**DOI:** 10.1007/s11136-025-03989-w

**Published:** 2025-05-17

**Authors:** Tessa Peasgood, Nancy Devlin, Kristina Ludwig, Ole Marten, Emily McDool, Paul Schneider, Koonal Shah, Clara Mukuria

**Affiliations:** 1 School of Medicine and Population Health, University of Sheffield, Sheffield, UK; 2 School of Population and Global Health, University of Melbourne, Melbourne, Australia; 3 School of Public Health, Universitat Bielefeld, Bielefeld, Germany; 4 Valorem Health, Bochum, Germany; 5 National Institute for Health and Care Excellence, London, UK

**Keywords:** Online elicitation of Personal Utility Functions (OPUF), Cognitive debrief, Think-aloud, EQ-HWB-S, Valuation health, Interview, Online data collection

## Abstract

**Purpose:**

Online elicitation of Personal Utility Functions (OPUF) is an innovative approach to valuing health states. OPUF uses a combination of ranking, swing-weighting, levels-rating and anchoring dead tasks. Little is known about how participants interpret and engage with OPUF tasks. This study aimed to address this gap.

**Method:**

Cognitive debrief interviews, which included ‘think-aloud’ and probing questions, were undertaken in the UK to understand how members of the public engage with OPUF when used to value the EQ-HWB-S (EQ Health and Wellbeing Short version). Coding drew upon a Framework approach, with final codes including an assessment of how participants engaged with each of the five OPUF tasks based on whether (1) they completed as expected, (2) minor concerns were identified or (3) major concerns were identified. The presence of major concerns was judged to undermine the validity of responses.

**Results:**

All 27 interviews were identified to have at least minor concern and 18 (67%) were identified as having major concerns. Major concerns were identified in four of the tasks: ranking (in 19% of interviews), swing-weighting (30%), levels-rating (56%), anchoring dead task (48%). Older participants were more likely to have major errors.

**Conclusion:**

Think-aloud and probing interviews with 27 participants completing the OPUF identified multiple concerns, to the extent that their data is unlikely to be a valid reflection of their preferences. The extent of concerns identified here suggests the need for interviewer led data collection within OPUF to ensure data quality.

**Supplementary Information:**

The online version contains supplementary material available at 10.1007/s11136-025-03989-w.

## Introduction

Healthcare decision-making can be informed by economic analysis, which assesses treatment outcomes using quality adjusted life years (QALYs). QALYs combine life expectancy with health-related quality of life (HRQoL), using a scale where 1 represents full HRQoL and 0 represents dead [1]. Generic HRQoL measures, paired with value sets based on public or patient preferences, often generate these values [2]. The value sets are derived through valuation studies using methods like time trade-off (TTO). These studies typically involve large samples (e.g. n = 1000 [3]) to value subsets of HRQoL states described by the measure [2]. Responses are modelled to estimate partial values for each dimension-level coefficient, forming the value set. This approach is called ‘decompositional’ because the values for the entire HRQoL states are broken down into the estimated influence of each dimension-level on the value score.

The Personal Utility Functions (PUF) method, developed by Devlin et al. [4], is a preference elicitation technique originally used in face-to-face interviews to encourage reflection and deliberation. Schneider et al. [5] adapted it for online self-completion surveys creating the Online elicitation of Personal Utility Functions (OPUF). OPUF directly elicits partial values for each level in each dimension by guiding participants through a series of steps. These partial values are combined to determine the values for any state described by a measure.

OPUF uses swing-weighting, a method from Multi-Criterial Decision Analysis (MCDA), to determine the relative importance of different attributes. In this approach respondents compare the impact of improving an attribute from its worst to its best level (a “swing”) against similar swings in other attributes [6]. Preferences elicited using swing-weighting are consistent with axioms that describe rational choices (i.e., completeness, transitivity and independence). The weight assigned to each attribute acts as a scaling factor, allowing scores on one attribute to be meaningfully compared with scores on others [7]. The shape of the partial value function in MCDA may be linear or empirically derived using methods like direct rating (Visual Analogue Scale (VAS) questions) or repeated bisection, where participants judge midpoints between worst and best levels [6, 8]. OPUF uses a VAS approach and assumes an additive function for the overall value, as common in MCDA [9]. For QALY calculations, values must be anchored to a 0–1 scale [10]; OPUF uses VAS for this step.

The compositional OPUF method requires fewer participants to estimate an aggregate mean value set [10], and allows for individual-level value sets since each participant values all the dimensions and response levels. This contrasts with decompositional methods, where participants value only a subset of states. Consequently, interest in OPUF has grown as a method for valuing measures online with smaller samples [5] and with patients [11].

OPUF can be applied to any measure. This study focuses on the EQ-HWB-S (EQ Health and Wellbeing Short version), a new generic measure covering health, social care and carer related quality of life [12]. The EQ-HWB-S covers nine domains: mobility, daily activity, exhaustion, loneliness, cognition, anxiety, sadness/depression, control, and pain. Each domain has one question with five response level options. A feasibility study using TTO and Discrete Choice Experiment (DCE) [13] showed these methods are viable but cognitively demanding due to the measure’s many items and levels. OPUF offers a promising alternative and has been tested in a feasibility study [14]. However, little is known about how participants engage with OPUF tasks or whether resulting values are valid and anchored to the 0 to 1 QALY scale. This study aims to address these gaps.

## Methods

Cognitive debrief interviews [15] were conducted to understand how the public interprets OPUF tasks when valuing EQ-HWB-S (see S1 for details of EQ-HWB-S) and to explore participants’ thought processes during task completion. Using think-aloud and probing responses, we aimed to assess the validity of the OPUF method. The think-aloud methodology was chosen for its ability to provide the best evidence on thought processes [16]. These approaches have been used successfully in understanding how individuals make decisions when completing health-state valuation studies [17–21].

Ethical approval was granted by the University of Sheffield (050470). A COREQ checklist is in S6. Interviews were conducted online with UK adults recruited via Prolific, targeting a gender balance and three to four participants in each of six age categories ranging from under 30 to over 70. Data saturation was expected at around 20 interviews [22]. Consent was obtained through an online survey before interviews. Survey steps are described in Table 1, and a test version is available here: https://valorem.health/eqen-demo.

**Table 1:** Survey and OPUF steps

Step	Description of participant activities	Supplementary figures
1	Complete the EQ-HWB-S, based on their own health and wellbeing in the last 7 days, and additionally, an adapted version of the EQ VAS. This both acts as a warmup and familiarises participants with the EQ-HWB-S	
2	A text box appears on the screen informing participants that although the previous part of the survey was based on their own current health, the next questions relate to problems they may not have experienced	
3	Complete a dimension **ranking task** in which participants are asked to consider each of the nine health and wellbeing problems in EQ-HWB-S at their worst level (e.g. Getting around: Unable; Feeling anxious: Most or all of the time) and imagine experiencing that problem but no other problem with health and wellbeing. They are then asked to rank each health and wellbeing problem *from worst (first) to least bad (last)*’ in a list format by dragging dimensions from left to right. The ranking box does not allow dimensions to be equally ranked	S3.1
4	Complete a dimension **swing-weighting task** in which participants are asked to compare and rate improvements in the nine dimensions of health and wellbeing. They are asked to consider an improvement from the bottom level to the best level in the dimension ranked as worst in the previous task, e.g. moving from ‘Getting around: unable’ to ‘Getting around: no problems. Respondents are told that this improvement has a rating of 100 points and is to be used as a reference to compare to the other improvements. The instructions note that 0 points implies the improvement is not important, 100 points is the most important improvement and between 1 to 99 points the improvement is important to them, but not as important as the most important improvement Participants may score more than one dimension swing at 100	S3.2
5	Complete a **levels-rating task** for each dimension. The task begins by showing the participant that the best level of a randomly selected dimension has a score of 100 and the worse level has a score of 0. They are asked to score the three intermediate levels using a slider starting with the second-best level. The best level is shown first using a greyed-out slider showing a score of 100. If participants score levels such that a worse or more severe level has a higher score than a better level a feedback box appears on the screen stating “*Are you sure about these ratings? Your response means that a level with more severe issues has an equal or better rating than a level with less severe issues. Do you want to check your ratings again or continue to the next page*?”	S3.3
6	Complete a **pairwise choice task** in which they are asked which they think is best, Scenario A which is the worst state described by the EQ-HWB-S, or Scenario B which is described as ‘Being dead.’	S3.4
7	Complete an **anchoring dead task** in which they assign a value to the preferred state from the previous task e.g. if they selected ‘Being dead’ in the previous task, the anchoring dead task asks them to locate the position of dead on a VAS scale anchored at 100 (labelled as ‘no health or wellbeing problems’) and 0 (labelled as ‘Scenario A’, and also linked to the description of the state on the screen by an arrow)	S3.5
8	Complete demographic and feedback questions	

OPUF task names are highlighted in bold

### Data collection

At the start of the interview, the interviewer demonstrated the ‘think-aloud’ process before sharing the survey link. Participants shared their screens while completing the survey; if this was not possible the interviewer shared it instead. Although conducted as interviews, participants self-completed tasks without guidance to simulate a self-complete environment. Interviews were conducted via Google Meet and lasted no more than one hour to meet participant expectations.

A topic guide was developed and tested by three researchers (CM, TP, EM) with input from the team (see S2). Two researchers (CM, TP) conducted the remaining interviews. Interviews were video recorded and automatically transcribed. Field notes captured technical issues and observations. Interviewers edited transcripts and added emotional reactions.

Participants were asked their views on using OPUF to inform decision-making. This data, however, is not analysed due to its limited depth. This may have been because the prompts came at the end of a long interview or were too open-ended, with participants agreeing with reasonable-sounding statements.

The researchers conducting the interviews met regularly throughout data collection to discuss interviews and data saturation. The target sample was adjusted to include more older participants where new themes were arising.

### Data analysis

Data analysis followed a Framework approach [23]. After familiarising with the data, the first three transcripts were coded independently by the three interviewers and the coding was reviewed collaboratively to develop an initial framework. Coding ensured consistency for errors and included deductive (drawing on study aims) and inductive themes. The framework was refined after dual coding two more transcripts (see framework in S4.1). Four additional transcripts were dual coded. Two researchers (CM and TP) independently coded the remaining interviews. Revisions continued as new codes emerged. Transcripts were coded in Microsoft Word and a framework matrix in Excel was used to summarised errors and extract relevant quotes. Findings are presented based on concerns identified in each OPUF task, focusing on the frequency of each concern to highlight recurring issues.

Codes evaluated how participants engaged with the tasks based on whether they (1) completed as expected, (2) had minor concerns, including (2a) common issues in health state valuation, (2b) interface-related problems, or (2c) issues with EQ-HWB-S, or (3) had major concerns suggesting lack of validity. Judgement on concerns were made collaboratively using criteria in Table 2.

**Table 2:** Criteria for coding error categories

Coding level	Criteria applied
Level 1: Task completed as expected	Based on our expectations of the task [4] [5] the interview data^*^ suggests the task was understood and completed as expected
Level 2a: Minor concern common to other health state valuation tasks	Interview data suggests a misunderstanding that has been identified in other valuation approaches such as TTO or DCE e.g. assuming that pain can be alleviated through medication; incorporating interactions between dimensions when rating each dimension separately
Level 2b: Minor concern which could be addressed within OPUF interface	Interview data suggests a confusion or difficulty which participants either self-correct or which could potentially be corrected with modifications to the interface. Data may still be a valid reflection of preferences
Level 2c: Minor concern which arose in relation to the EQ-HWB-S descriptor	Interview data suggests a problem with item wording or response choices which may not arise with another instrument
Level 3: Major concern which undermines the validity of the response	Interview data identifies a misunderstanding such that responses could not reflect preferences e.g. reversing the scales used, interpreting the scales as measuring something different to their intended meaning

^*^Interview data includes think-aloud response to interviewer prompts, and observations of participants interacting with the OPUF interface

Each task was prefixed in the coding: Ranking (R), Swing-weighting (S), Levels-rating (L), Pairwise choice (C), and Anchoring dead (D). This was paired with the 1 to 3 level concern codes. Individual concerns were numbered consecutively as identified (e.g. R3.1 refers to the first major concern identified in the Ranking task, R3.2 refers to the second major concern identified in the Ranking task etc.). This coding serves two purposes, firstly enabling the reader to identify the full quotes in the supplementary material and secondly, to communicate the assessment of concern severity for each issue raised.

## Results

### Participants

One-to-one interviews were completed with 27 participants between August and September 2023. Participants ranged in age from 22 to 79 years, 14 (52%) female, 16 (59%) holding a degree or equivalent and 14 (52%) reporting a long-standing illness. Further details are shown in Table S4.4.

### Summary of concerns

**Fig. 1:**
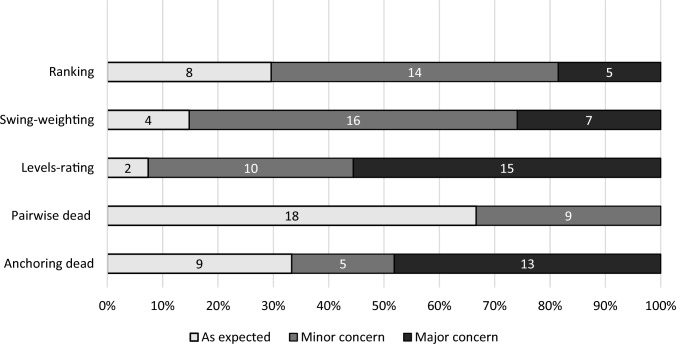
The most serious level of concern identified for each OPUF task for each interview (n = 27)

Concerns were identified in all five tasks, with one to 14 sub-categories coded. Figure 1 shows engagement with the five tasks based on the highest concern for each participant (i.e. major concerns counted over minor ones). The pairwise choice task had the fewest concerns, with 67% (n = 18) completing it as expected and no major concerns identified. However, other tasks showed high rates of both minor and major concerns. Over half of participants (56%, n = 15) had major issues with the levels-rating task, and nearly half (48%, n = 13) had major issues in the anchoring dead task. In the swing-weighting task, most of the participants had minor concerns (59%, n = 16).

All participants had at least one minor or major concern flagged. Nine participants (33%) had only minor concerns, while 18 (67%) had at least one major concern. Seven (26%) had major concerns in three or more tasks.

An age effect was seen when comparing participants aged 50 and above (n = 13) to those younger (n = 14). Older participants were more likely to have major concerns in the swing-weighting task (36% vs 14%), levels-rating task (64% vs 43%) and anchoring dead task (57% vs 36%), though differences were small due to sample size. Conversely, older participants were more likely to complete the pairwise choice task as expected (85% vs 50%).

In the following sections, we describe how participants approached each task, highlighting major and minor concerns. We report the number of interviews with each concern (also shown visually in S3.6 to S3.10). The text below links to codes from Table S3.2 showing example quotes. Quotes are assigned a participant/interviewer identifier (e.g. M1830_TP9) that represents participant gender (M/F), age (18–30/30–39/40–49/50–59/60–69/70 and above), interviewer (CM/TP/EM), and interview number.

### Ranking

Most participants understood the ranking task, though minor concerns arose in comprehending what was being valued. Some participants assumed that intervention to alleviate the problem would be possible (n = 6, R2a.1) while others assumed interactions between dimensions (n = 12, R2a.2); Participants referred to interactions between dimensions in EQ-HWB-S and dimensions not included, such as “*how somebody is managing the condition*” (M1830_TP9).

Three participants ranked dimensions based on their current health (R3.1), despite reading the pop-up text explaining the shift from personal experience to hypothetical states. Others initially made this error but self-corrected (so were not coded as having an error). The gap between current health and the hypothetical worse state made this error more apparent for some.

Two participants reversed the order, placing the worst dimension at the bottom (R3.2). This was coded as a major concern and is problematic because the swing-weighting task uses the top-ranked dimension as an anchor.

### Swing-weighting

Only four participants completed the swing-weighting task as expected. As in the previous task, some assumed interactions (n = 9, S2a.1) and that intervention was possible (n = 4, S2a.5).

Four participants either did not realise they could score multiple dimensions at 100 or felt doing so would be inconsistent with the previous task (S2b.11). Three used the scale to represent the likelihood of a move to having no problems in each dimension (S2b.6), considering scenarios like “*a hundred improvements*” in loneliness or moving from “*using a wheelchair to suddenly being 25 again*” (M5059_CM0) as improbable.

Eight participants showed no indication of scoring an improvement *relative* to the improvement in the most important dimension as required by the task (S2b.9). Two tried to adjust scores on the reference dimensions (S2b.12), and two assigned 100 to most dimensions without clear rationale (S2b.4).

Two participants needed assistance to complete the task (S2b.2) while four (S2b.10) completed independently after initial confusion. One revised their decision when probed (S2b.13). For some (n = 2, S2c.3), confusion arose from the EQ-HWB-S control item, where interpreting ‘lack of control none of the time’ was difficult. Others (n = 3, S2c.7) did not view the top level as the most desirable, reasoning that some problems with anxiety and sadness were “*perfectly normal human feelings*” (F6069_CM2).

Seven participants were classified as having major concerns: two answered based on their current health (S3.1), four scored 100 believing it represented complete (100%) improvement (S3.2), and two misunderstood the scale entirely (S3.3).

### Levels-rating

The levels-rating task was challenging for many participants, with only two completing it without concerns. One participant assumed intervention was possible (L2a.9), as in earlier tasks. Some participants struggled because they did not clearly distinguish between the EQ-HWB-S descriptors, especially “only occasionally” and “sometimes” (n = 4, L2c.1). Others were confused by the numerical scale for the frequency response options assuming it was asking about an interpretation of the meaning of each level, noting “*if I say I’m feeling lonely sometimes am I actually feeling lonely like 50% of the time?”* (F1830_TP13).

Eleven participants tried to move the fixed anchors (L2b.6), suggesting they had not fully engaged with instructions. One participant tried to move the bottom anchor to align to the score they gave the dimension in the previous swing-weighting task.

In probing responses, some interviews explored whether focusing on the relative differences between levels or starting from the bottom (worst to second-worst level) instead of the top (best to second-best level) would affect interpretation. Four participant’s responses were not robust to these perspective changes (L2b.7) and two more changed their responses during discussion (L2b.10).

The scale direction caused confusion, with some participants feeling the 0–100 scale had reversed from the previous task. Seven participants were too confused to complete the task independently (L2b.3). Others initially struggled but self-corrected or adjusted their responses after the feedback box flagged the issue (n = 4, L2b.4). Confusion with the scale direction led some participants to answer correctly at first but incorrectly in subsequent level-rating tasks (n = 6, L3.4). For one, this shift was triggered by the pain dimension which they perceived as substantially different to other dimensions. Others (n = 9, L3.2 and L3.2a) remained confused about the scale direction: five paused the survey after the feedback box and asked for help, while four ignored the feedback box and continued.

As in earlier tasks, some participants answered based on how closely the levels reflected their current experience (n = 3, L3.1). Additionally, one gave a score of 100, interpreting it as a 100% improvement from each level, reasoning also seen in the swing-weighting task (L3.3).

### Pairwise choice

The pairwise choice task was generally completed easily and as expected by most participants (n = 18). However, transitioning to the screen where “being dead” held a dominant position often prompted reactions like nervous laughter or surprise. Some participants interpreted the question as addressing their views on euthanasia (n = 5, C2a.1). As in other tasks, some assumed intervention might be possible (n = 4, C2a.2).

### Anchoring dead

Only nine participants completed the anchoring dead task as expected. As in the previous task, one participant thought it was about their views on euthanasia (D2a.2). Many found the task unclear, particularly when rating ‘dead’, which occurred when participants said being dead was better than the worst EQ-HWB-S state. A few (n = 3) were unable to complete the task (D3.2). Others re-interpreted the question, answering based on where they would personally like to be on the scale (n = 3, D3.3), relating it to personality (n = 1, D3.6), their current health (n = 1, D3.4) or how long they would endure a poor state before wanting to die (n = 1, D3.7). Some interpreted the scale as a percentage difference to the bottom of the scale (n = 5, D3.1) scoring 100 because their preferred state was 100% better than the not preferred state.

Additionally, one participant used the scale for ordinal preference only (D2b.1), one changed their response after probing (D2b.5), and two answered correctly after initial confusion (D2b.3). One participant set “dead” at zero, reasoning that “dead” has no quality of life (D2b.4). Labelling also caused confusion with some (n = 3, D3.5) scoring “dead” at 100 equated it with having no health or wellbeing problems, as described for the top anchor.

### Engagement

#### Reading instructions

Each task was coded based on how thoroughly participants read instructions. Due to screen-sharing difficulties and silent reading, not all interviews and tasks were classified, so we do not report reading frequency. We identified several patterns: (i) participants read all instructions, including ‘additional details’ tabs; (ii) some missed or ignored the additional details *(“Yeah, I mean I read through this and this three times and I just completely missed the further details*” (F1830_TP11), (iii) some only read the bolded text, and (iv) some attempted to answer before reading instructions. Many participants, particularly younger ones, interacted with the interface before fully reading instructions or deciding on answers. For example, in the final anchoring dead task, some immediately tested whether the top slider moved; in the ranking task, some immediately dragged dimensions across; and in the levels-rating task some set the levels at 25, 50, 75 before deciding their responses.

#### Confusion with scales

A 0–100 scale is used in three of the five tasks. Participants also completed a 0–100 VAS for their own health and wellbeing before the first task. While 100 represents good in all cases, the mixed use across tasks caused confusion, especially in the levels-rating task. Participants noted that ‘no problems’ intuitively felt like it should be zero, as one explained, "*I think of nothing like no difficulty as a zero.*" (F6069_CM2) (see example quotes in S4.3).

#### Insufficient information

In some interviews, participants requested further clarification, suggesting insufficient information had been given. This included questions about the anchoring dead task which lacked a time period and requests for more information on health states where dimensions are assessed in isolation. Participants noted, for example, that it “*doesn’t say your mental faculties are affected*.” (M4049_CM3). Many participants engaged in tasks despite limited understanding with some adapting the tasks to complete them, such as reinterpreting scales or adding details, like assuming the presence of a carer.

#### Issues valuing EQ-HWB-S

Some problems stemmed from difficulties interpreting the abbreviated EQ-HWB-S items not the OPUF tasks themselves. Ambiguity arose from double-barrelled items and difficulty interpreting response levels; for example, one participant noted “*I was sort of chasing around, trying to decide—What does occasionally mean?*” (M70 + _TP3) Interpretation was likely worsened by participants dropping the ‘only’ from “only occasionally” when considering this level.

#### Feedback and suggested improvements

Participants’ views of the survey ranged from finding it “*straightforward*” (M4049_CM3) to challenging, with one stating it required “*above average intelligence*” to understand (M70 + _TP8). Some who reported they found it easy did so despite having clear difficulties. For example, one participant noted “*Yeah, all really simple and intuitive. Nothing confusing*” yet earlier stated “*I’ve got the wrong way. Sorry, I’m not right. So, everything has some difficulty … No difficulty has a value of 100. But then, it feels backwards.*” (M4049_CM4).

Some described the accuracy as artificial, stating the numbers “*feel a bit arbitrary.*” (F1830_TP11) Others reflected on using multiples of ten in their responses, saying “*I always tend to go for tens anyway.*” (M70 + _TP2).

Participants suggested improvements, such as encouraging reading of the optional information, providing more context on the survey’s purpose as motivation, and reversing the levels-rating task formatting so that no problems appears at the bottom.

## Discussion

This study aimed to explore how participants interpret OPUF tasks and assess whether their responses can generate values anchored to the QALY scale. Major concerns, interpreted as undermining the validity of the data, were identified in four of the five tasks and in 18 (67%) interviews. Major concerns were often related to how the scales were interpreted, with changes in the meaning of scales between tasks creating confusion.

A few participants, mostly older, tried to complete tasks in relation to their current health. This does not produce valid responses though this may not be obvious when assessing quantitative data.

Within standard OPUF analysis, data are dropped if a participant reverses the scale in the levels-rating questions for at least three dimensions or if the anchoring dead task has an answer of 100. For example, in a UK valuation of EQ-HWB-S, 5% of the sample were dropped for answering 100 in the anchoring dead task; in the German version of the survey for the same study, participants were automatically dropped if they provided three implausible responses to the levels-rating task [14]. These errors were identified in participants who were not considered to be disengaged by the interviewers. Other participants had values that reflected something different to what was intended in the task, yet this would not be flagged by these checks.

Some of the concerns identified also occur in other valuation methods and were classed as minor because the difficulty may rest with valuation itself rather than the OPUF method. Concerns relating to assuming that the state may change are equally problematic for other methods [18, 24]. However, the implications may have been underplayed to date as the assumption of symptom alleviation was made for some dimensions (e.g. pain (L2a.9), anxiety (C2a.2), loneliness (R2a.1), sad/depressed (S2a.5) see Table S3.2 for example quotes), but not all, impacting relative weights. Assumptions about interactions between dimensions also occur in other valuation methods, however, they are potentially more problematic in OPUF. Decompositional methods can incorporate interactions but within the compositional approach designing tasks to capture interactions is particularly challenging [4]. That said, modelling within the decomposition approach rarely incorporates interactions and is unlikely to be feasible for long instruments [25].

Swing-weighting can suffer from anchoring bias arising from setting the starting question at 100 points [8] leading to overestimation of unimportant changes [26]. In this study, the swing-weighting, levels-rating and anchoring dead tasks each had two anchors, although attention is drawn to the top (100) anchor. We found evidence of participants being influenced by the anchors. Swing-weighting can also be impacted by equivalising bias, where respondents rate all domain swings the same (e.g. 100 for all) [8] which also flattens the distinction between domains [26]. Some participants gave the same score to multiple dimensions in the swing-weighting task, but it is unclear whether this reflects a bias, a real preference, or a different problem such as not referring to the reference dimension. Two studies comparing DCE with swing-weighting (both online) found that the relative importance of the attributes was more evenly distributed across attributes in swing-weighting than DCE [27, 28].

The problems identified in the levels-rating task may suggest the need to reconsider the ‘direct rating’ approach for this task. This approach has been criticised for having “limited rigour” within MCDA as it “conflates assessment of the performance with the strength-of-preference for that performance” [29]. Some evidence of this is seen here, such as participants who interpreted the slider as a means of explaining the meaning of response options and those who immediately responded with 25:50:75 for each of the levels. This suggests they are responding based on their interpretation of the response level, rather than what they think it would be like to live in a state described by the level.

Other MCDA studies have identified challenges with respondent completion of swing-weighting tasks online. For example, in a wastewater preference elicitation task online, 95% of the sample was identified as not adhering to swing-weighting instructions [30].

Problems with the OPUF method occurred whilst valuing the EQ-HWB-S. While some difficulties may have been exacerbated by the measure’s length (requiring more task repetition) and item wording many of the problems arose at the start of tasks, and with items similar to other measures (e.g. pain).

Online engagement may have influenced responses, as some participants did not read information and viewed the task before deciding whether to read the question fully. Additionally, some participants failed to digest information even after reading it. These findings offer valuable insights for unsupervised online valuation beyond the use of OPUF.

One solution to address the issues identified could be interviewer-led data collection, with interviewers adopting a proactive role, as in the original PUF approach [4]. The use of interviewers is widely adopted in Time Trade Off data collection due to task complexity, although can lead to interviewer-dependent values [31]. Future protocols for OPUF could include data quality control procedures and ensure interviewer compliance to avoid any potential interviewer effects. Adjusting how information is presented in OPUF could reduce confusion and ensure all relevant details are visible. Using feedback messages may also enhance engagement, and incorporating feedback across all tasks could be beneficial. Any modifications of the approach would require further qualitative and quantitative testing, including test–retest [32] and testing for interviewer effects.

### Strengths and limitations

Participants were recruited through Prolific, an online survey company, which may limit representativeness. However, recent OPUF studies also used Prolific [14] making it suitable for accessing participants’ understanding.

As with any cognitive debrief interview, the act of interviewing can influence participant behaviour [33]. While participants were asked to behave as if we were not present, interviewers occasionally intervened to prevent prolonged struggles. It is unclear whether participants would have resolved confusions independently.

The study combined think-aloud and probing methods, which helped identify issues that might have been missed with one approach. The use of multiple researchers for interviews and coding, with similar issues identified, adds validation to the findings. (see S5 for a reflection on potential researcher bias). The systematic counting of concerns and their severity was a rigorous method for reporting data validity concerns, though the small sample size limits the ability to present quantitative results. This rigorous cognitive debrief was able to identify and evidence misinterpretations that would not have been identified through asking respondents if they understood tasks, or through assessing their quantitative responses. As such, is it a useful step in the development of health state valuation methods.

## Conclusion

Think-aloud and probing interviews with 27 participants completing the OPUF revealed multiple concerns, suggesting the data may not reflect true preferences, particularly for older participants. The interviews also showed tasks being completed accurately with participants making considered judgements. While participants suggested ways to improve comprehension, many, even under observation, did not fully read instructions, making it unlikely that modified instructions alone would resolve all issues. The extent of concerns identified indicates the need for interviewer-led data collection to ensure data quality. Given the advantage of the OPUF approach for health state valuation in terms of sample size, further development of the approach is still warranted.

## Supplementary Information

Below is the link to the electronic supplementary material.

Supplementary file1 (DOCX 1158 KB)
