# Supplementary material for: How well do participants understand the questions asked in the Online Personal Utility Functions (OPUF) approach? A cognitive debrief of the EQ-HWB-S (EQ Health and Wellbeing Short version) valuation
Source: Qual Life Res. 2025 May 17;34(8):2269–78. doi: 10.1007/s11136-025-03989-w (PMC12274140; doi:10.1007/s11136-025-03989-w)
Supplement: Supplementary file 1 — Supplementary file1 (DOCX 1158 KB) [file 11136_2025_3989_MOESM1_ESM.docx]

Supplementary material

**Title: How well do participants understand the questions asked in the Online Personal Utility**

**Function (OPUF) approach? A cognitive debrief of the EQ-HWB-S (EQ Health and Wellbeing Short version) valuation.**

Contents

[1 S1 Details of EQ-HWB-S 2](#_Toc186887313)

[2 S2 Interview topic guide for ‘think aloud’ interviews exploring OPUF 2](#_Toc186887314)

[3 Supplementary Figures 7](#_Toc186887315)

[3.1 Ranking task 8](#_Toc186887316)

[3.2 Swing-weighting task 9](#_Toc186887317)

[3.3 Level-rating task 10](#_Toc186887318)

[3.4 Pairwise choice task 10](#_Toc186887319)

[3.5 Anchoring dead task 11](#_Toc186887320)

[3.6 Interviews with identified errors in Ranking task 12](#_Toc186887321)

[3.7 Interviews with identified errors in Swing-weighting task 13](#_Toc186887322)

[3.8 Interviews with identified errors in Levels-rating task 14](#_Toc186887323)

[3.9 Interviews with identified errors in Pairwise choice task 15](#_Toc186887324)

[3.10 Interviews with identified errors in Anchoring dead task 16](#_Toc186887325)

[4 Supplementary Tables 17](#_Toc186887326)

[4.1 Table S4.1 Coding framework 17](#_Toc186887327)

[4.2 Table S4.2 Frequency of concerns identified and example quotes 23](#_Toc186887328)

[4.3 Table S4.3 Example quotes for other themes 35](#_Toc186887329)

[4.4 Table S4.4 Participant details 38](#_Toc186887330)

[5. S5 Reflection on researcher biases 38](#_Toc186887331)

# S1 Details of EQ-HWB-S

The EQ-HWB-S has nine dimensions: mobility, daily activity, exhaustion, loneliness, cognition (trouble concentrating or thinking clearly), anxiety, sadness/depression, control, and pain, each with five response level options (Brazier et al., 2022). Each dimension has a single item (or question). The mobility and daily activity item levels are expressed in terms of difficulty: no, slight, some, a lot, unable. The pain item is expressed in terms of severity: no, mild, moderate, severe, very severe. The rest of the items have frequency levels: none, only occasionally, sometimes, often, most or all of the time. It has a seven-day recall period.

The EQ-HWB instrument has two versions, a long 25 item version (referred to as EQ-HWB) and a short 9 item version (referred to as EQ-HWB-S), which is the one used in this study. The long version includes the 9 items from the S along with additional items such as hearing, vision, sleep, memory, frustration, coping, discomfort, support from others, and safety.

Both instruments are, at the time of writing, considered to be in an ‘experimental’ stage by the EuroQol group. This means they are available to researchers for testing and may be subject to minor future amendments.

# S2 Interview topic guide for ‘think aloud’ interviews exploring OPUF

**1. Welcome**

Interviewer introduces themselves and asks questions unrelated to the research to put participant at ease and establish rapport:

- *How has your day been?*
- *Where are you calling in from?*
- *Etc.*

**2. Introduction and checking consent**

Consent process – if not done before

Interviewer will give a brief recap on the research aims

*We’re going to be looking at questions which ask you about different aspects of health and wellbeing and how important you think they are, whether or not you’ve experienced them.*

*I just want to remind you that you are free to withdraw at any time – just let me know - and you do not have to offer a reason.*

*We will record the interview – which will include the sound and the camera – this is so we can re-listen to it afterwards as it is difficult for me to take notes while I’m interviewing. Some of the things you say during the interview may be used in a quote for our report, but it won’t be possible to identify you.*

*Are you happy to continue?*

**[Start recording]**

**3. Introduction to think aloud**

*We’d really like to understand what you are thinking when you see the questions in this survey so we will do the interview in two parts, the first where you complete the survey and the second part where we ask you some questions.*

*In the first part, I’d like you to complete some of the survey as if you were completing it on your own but while you answer the questions, I would like you to talk me through your thought processes – just saying whatever is coming into your head as you see each question. There are no right or wrong answers or thoughts here, we just want to know what thoughts people have when working through the survey themselves.*

*We tend to call this ‘thinking aloud’. I’ll give you example of what we mean by ‘thinking aloud’. Imagine I was answering a question on how important green space was to me on a scale of 1 to 10 where 1 is not important and 10 is very important while thinking aloud – I might say something like this….*

*“I wonder what green space means – is that like my garden or any green space? I guess it’s really important – I like to look out the window and see a bit of green and to sit and have my lunch outside somewhere with some grass. It’s calming. Oh – 1 to 10 – I don’t know really it depends – I think a 7 cause it’s important but well it could be 6 or 5 I don’t really know how to compare it”*

*So – you see it doesn’t need to be particularly structured – it’s just saying the things that pop into your head as you read each question.*

[Note: interview to edit above as feels comfortable for them to say]

*For each question, I may prompt you to provide further details to help us understand how people reach specific answers.*

*In the second part of the interview, I will ask you additional questions about your views on the survey itself.*

3.1 EQ-HWB Questions

These questions are about your own health and wellbeing. Pease complete them without thinking aloud.

**4. Prompts/questions during the OPUF Survey**

*I’m going to ask you to click on the link in that you received and share your screen and we’ll see the survey questions - Do you have any questions before we start?*

If participant has difficulties sharing the screen the interviewer will share their screen instead.

Interviewer will note down any time that the participant clicks ‘show detail’ in the survey.

If the participant asks for clarification prior to giving a response the interviewer will say:

*Before I say anything too much – can I just ask what you would have assumed or guessed if I wasn’t here – the reason I ask that is we want to know what people might do if they were working through the survey online by themselves.*

4.1 Ranking

Interviewer to prompt starting the ranking question and encourage participant to think aloud

- *Can you tell me a bit about what you are thinking now?*
- *Can you tell me why you put those two at the top?*
- *Can I just check – when you said XXXXX did you mean XXXX*

Note: need to take care not to lead participants. The questions from the interviewer should focus on being able to assess participant understanding of the task.

4.2 Importance

- *Can you tell me a bit about how you decided on that score?*
- *I see you chose X as the lowest scoring improvement – would you say it really wouldn’t be important?*
- *Do you think you wanted to spread out your responses?*
- *Did that feel this question was different to the previous question where you just put them in order? Were you thinking about similar things?*
- *Did you feel you could have given equal scores to more than one domain?*
- *Did you think the survey was wanting you to give a different order or the same order as the previous question?*
- *When you were thinking about improving one aspect at a time – say pain – what were you thinking about other aspects in your life?* [If further prompt needed…] *Did you think a life with extreme pain would have no problems in any other aspect or did you think that a life with extreme pain would have problems across other aspects too?*

Interviewer to flag any differences between the importance and ranking

*That’s interesting – when you did the ranking you thought X was more important – which question do you think is better for getting at your views?*

4.3 Levels

If the participant has not expressed how they are doing the task in their own words sufficiently, probe to check how they approached the task.

- *Can you tell me a bit about what you were thinking about when you chose those numbers?*
- *Did you come back to how you judged the most important improvement?*
- *Did this task make sense to you? ?*

4.4 Anchoring

- *Can you tell me what you were thinking about during this task?*
- *Why do you think the survey is asking this?*

4.5 Own values

Participant to be shown their own set of values.

Interviewer will point out a couple of things on the individual’s value set to help them with interpretation.

*e.g. – For your scores it is showing us that you considered having ‘extreme pain’ to be the most important problem.*

- *What do you think about these scores?*
  - *Do they feel in line with your views?*
  - *Do any of them surprise you?*
- *Do you think seeing your own scores like this tells you anything useful?*

4.6 Use in decision making

The interviewer will give a brief explanation of how scores are used in decision making.

*These type of scores could potentially be used to support decision making in health care. They could help understand how important you were likely to think different types of side effects of a possible treatment would be for example.*

*We could also combine lots of people’s views together. If we find that most people give a lot of importance to improvements in mobility compared to anxiety, then treatments that improve mobility would be seen as offering a more valuable benefit than treatments for anxiety – so decision makers may be willing to pay more for those treatments.*

- *How do you feel about your answers being used to support decision making - for yourself and for others?*

**5.0 Semi-structured discussion & debrief**

*This final part of the interview is more of a discussion where you get a chance to tell us what you thought of the survey and how people are likely to answer it.*

*Firstly can I ask a couple of things about the actual survey*

- *Were there any questions you found difficult to answer for any reason?*
- *Were there any questions where you felt you needed more instructions or information?*
- *Were there any questions where you felt you were just answering a bit randomly?*

*How did you find navigating through the survey online? [Online text, information, graphics, steps]*

*Is there anything else you would like to tell us about how you think we could improve the survey or about the survey in general?*

**6. Conclusion**

*Excellent that’s the end of the interview – thank you so much for talking through your views and how you answered those questions.*

*Thank you very much for your time, the information you have provided is extremely valuable and will help us to understand how people complete these type of surveys.*

*I hope you enjoy the rest of your day/evening*

*Nice to meet you, Etc.*

# Supplementary Figures

Supplementary Figure 1: OPUF tasks

| Ranking task 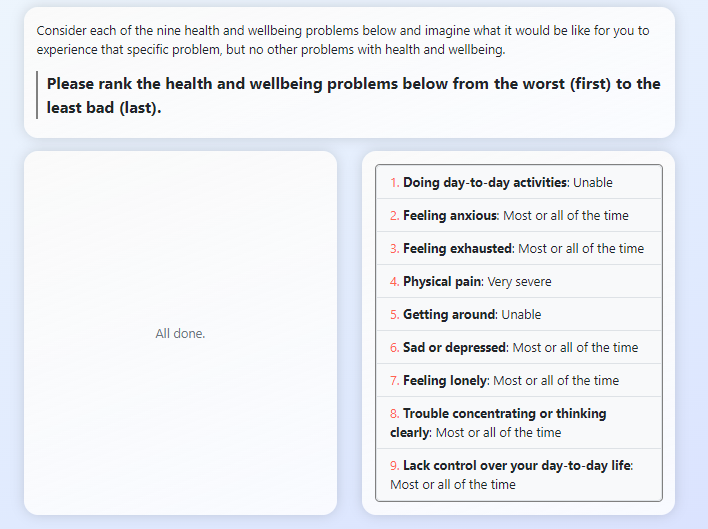 |
| --- |
| 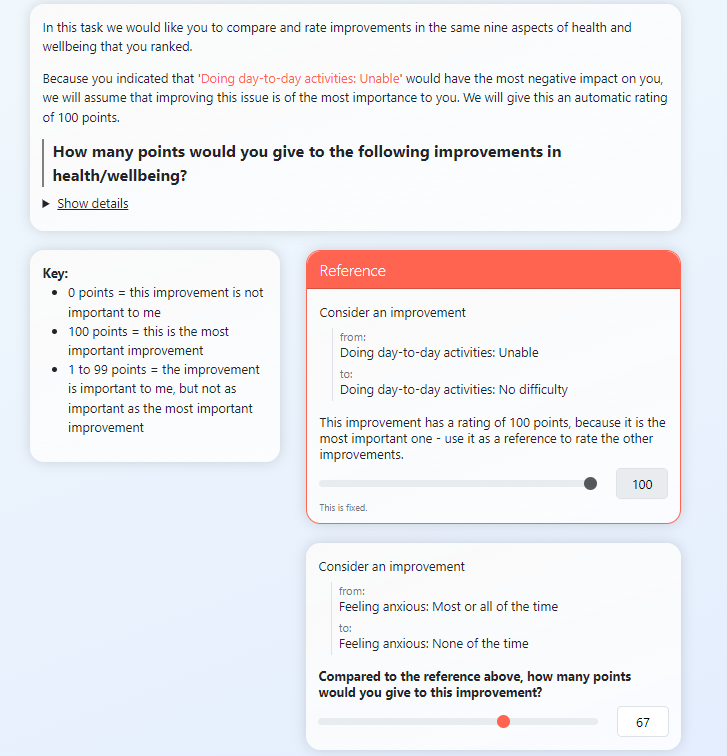Swing-weighting task |
| 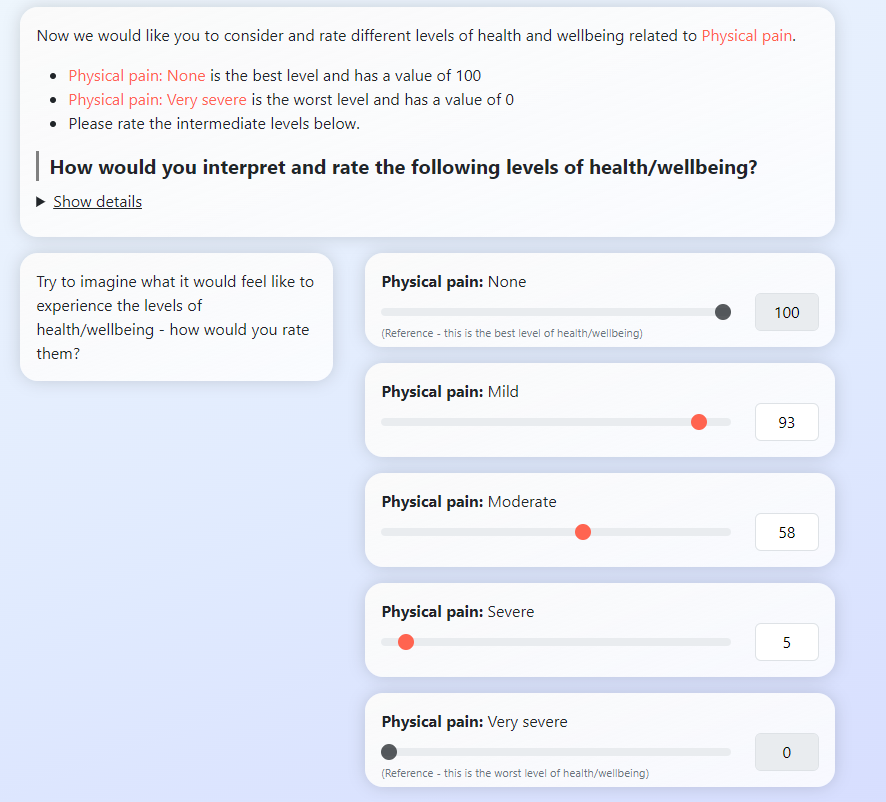Level-rating task |
| Pairwise choice task 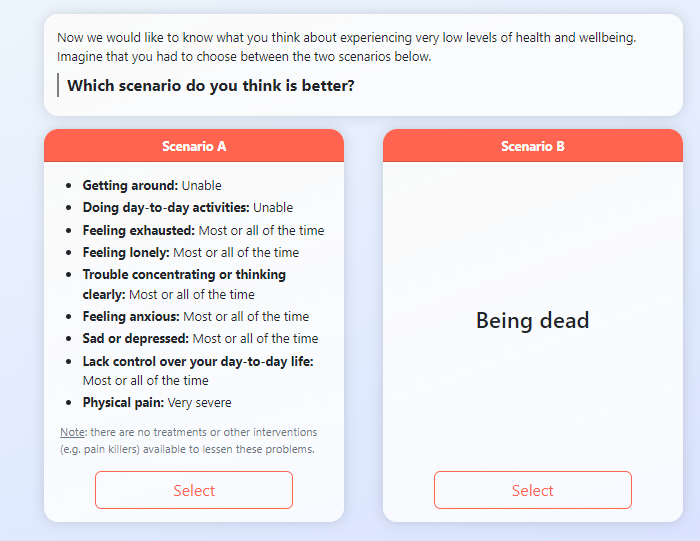 |
| Anchoring dead task 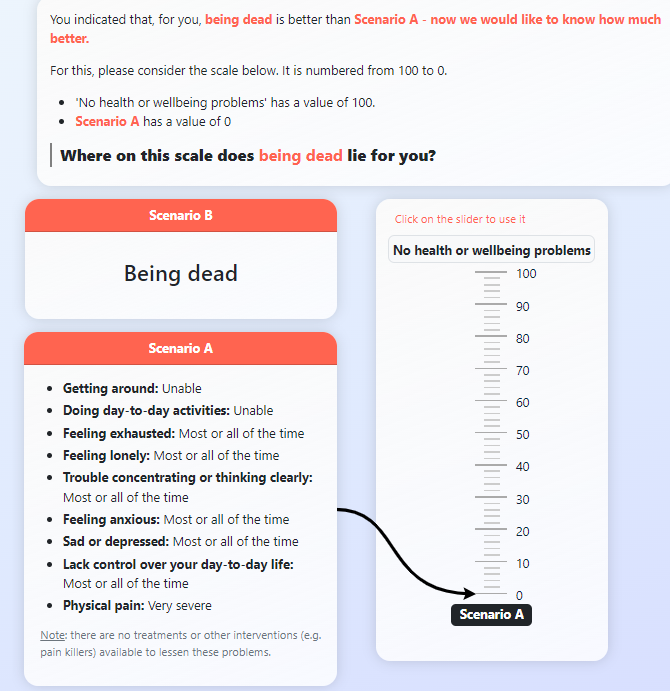 |

## Interviews with identified errors in Ranking task

Key: Level 2 = minor concern (**2a: concern common to health state valuation**, **2b: concern relating to OPUF task**, **2c: concern relating to EQ-HWB-S instrument**). **Level 3 = major concern**

## Interviews with identified errors in Swing-weighting task

Key: Level 2 = minor concern (**2a: concern common to health state valuation**, **2b: concern relating to OPUF task**, **2c: concern relating to EQ-HWB-S instrument**). **Level 3 = major concern**.

## Interviews with identified errors in Levels-rating task

Key: Level 2 = minor concern (**2a: concern common to health state valuation**, **2b: concern relating to OPUF task**, **2c: concern relating to EQ-HWB-S instrument**). **Level 3 = major concern**.

## Interviews with identified errors in Pairwise choice task

Key: Level 2 = minor concern (**2a: concern common to health state valuation**, **2b: concern relating to OPUF task**, **2c: concern relating to EQ-HWB-S instrument**). **Level 3 = major concern**.

## Interviews with identified errors in Anchoring dead task

Key: Level 2 = minor concern (**2a: concern common to health state valuation**, **2b: concern relating to OPUF task**, **2c: concern relating to EQ-HWB-S instrument**). **Level 3 = major concern**.

# Supplementary Tables

## Table S4.1 Coding framework

|  | **1 Interpreted as expected** | **2. Minor problems** | | | **3 Major problem** | **4 Issues arising** | **5 Strategies adopted** |
| --- | --- | --- | --- | --- | --- | --- | --- |
|  |  | **2a. Common to other valuation methods** | **2b. Relating to OPUF tasks** | **2c. Relating to EQ-HWB-S items or presentation** |  |  |  |
| **Ranking (R)** | R1 Interpret as expected | R2a.1 Assumes intervention possible to described state (e.g. took more rest) |  |  | R3.1 Order based on levels currently experienced |  |  |
|  |  | R2a.2 Assumes interactions between domains |  |  | R3.2 Ranking in the reverse order |  |  |
|  |  |  |  |  |  |  |  |
| **Swing weighting (S)** | S1 Interpret as expected | S2a.1 Assumes interactions between domains | S2b.2 Struggled to complete task independently | S2c.3 Difficulties with presentation of EQ-HWB descriptor e.g. no control/control double negative, control is vague | S3.1 Response based on levels currently experienced |  | SS |
|  |  | S2a.5 Assumes intervention possible to described state | S2b.4 Answers most at 100 without clear reasoning | S2c.7 Response based on having no problems in a domain not being ideal | S3.2 Scoring as 100 because the domain would be completely improved i.e. 100% or because you would choose to be at 100 for each |  |  |
|  |  |  | S2b.6 Response based on how likely having no problems would be in a domain |  | S3.3 Lack of understanding about 0 to 100 scale |  |  |
|  |  |  | S2b.9 Reference domain not referred to in rating or have problems interpreting the anchor |  |  |  |  |
|  |  |  | S2b.10 Confused initially but then interprets as expected |  |  |  |  |
|  |  |  | S2b.11 Assumes can't have more than one domain rated at 100 |  |  |  |  |
|  |  |  | S2b.12 Tries to score reference dimension |  |  |  |  |
|  |  |  | S2b.13 Changes answer in response to discussion |  |  |  |  |
|  |  |  |  |  |  |  |  |
| **Levels-rating (L)** | L1 Interpret as expected | L2.9 Assumes intervention possible | L2b.2 Confuses the levels of the slider with judgement of frequency | L2.1 EQ-HWB level descriptors are not distinguishable. | L3.1 Response based on levels currently experienced |  | LS |
|  |  |  | L2b.3 Struggled to complete task independently |  | L3.2 Interprets the 0-100 the wrong way round or confused by the 0 to 100 scale |  |  |
|  |  |  | L2b.4 Confused by order but self-corrects or after pop-up box |  | L3.2a Interprets the 0-100 the wrong way round or confused by the 0 to 100 scale AND ignores the pop-up error message |  |  |
|  |  |  | L2b.5 Uses the ends of the scale to highlight good/bad providing an ordinal preference |  | L3.3 Scores as 100 because the domain would be 100% improved |  |  |
|  |  |  | L2b.6 Tries to move fixed top or bottom |  | L3.4 Confused (based on verbal or data) after previously getting it correct. |  |  |
|  |  |  | L2b.7 Reference bias i.e. rates levels based on top or bottom levels |  |  |  |  |
|  |  |  | L2.10 Changes their answer in response to discussion |  |  |  |  |
|  |  |  |  |  |  |  |  |
| **Pairwise choice (C)** | C1 Interpreted as expected | C2a.1 Assumes question is about euthanasia |  |  |  | C4.1 Has done a DCE before | CS |
|  |  | C2a.2 Assumes change is possible (e.g. intervention) |  |  |  |  |  |
|  |  |  |  |  |  |  |  |
| **Anchoring dead (D)** | D1 Interpreted as expected | D2a.2 Assumes question is about euthanasia | D2b.1 Number given represents ordinal preference only |  | D3.1 Scores at 100 because it is the opposite to the worse state or 100% better than worse state |  | DS |
|  |  |  | D2b.3 Confused initially but then interprets as expected |  | D3.2 Unable to complete task |  |  |
|  |  |  | D2b.4 Dead at zero because dead has no quality of life |  | D3.3 Reinterprets the VAS to rate where they would want to be |  |  |
|  |  |  | D2b.5 Changes answer in response to discussion |  | D3.4 Reinterprets the VAS as own health |  |  |
|  |  |  |  |  | D3.5 Reinterprets the top of the scale in relation to dead i.e. when dead you have no health and wellbeing problems |  |  |
|  |  |  |  |  | D3.6 Misunderstanding the task e.g. thinking it was about personality |  |  |
|  |  |  |  |  | D3.7 Reinterprets scale to mean the proportion time someone would wish to stay alive in a very bad state |  |  |
|  |  |  |  |  |  |  |  |
| **Approach to task** | T1.1 Reads (aloud or allows enough time) all question text (including any additional details) before scrolling to answer | T2.1 Reads only statements in bold before scrolling to answer |  |  | T3.1 Does not read before trying to answer | T4.1 Reacted to dead task e.g. laughed, exclaimed etc. |  |
|  | T1.2 Reads (aloud or allows enough time) main question before scrolling to answer |  |  |  |  |  |  |
|  |  |  |  |  |  |  |  |
| **Valuation approach (V)** |  | V2.1 Interprets dimension or level by adding information because it's been stripped out e.g. assumes has carer | | |  |  |  |
|  |  | V2.2 Asks for meaning of term | | |  |  |  |
|  |  | V2.3 Interpretation of frequency level | | |  |  |  |
|  |  | V2.4 Missing time frame | | |  |  |  |
|  |  | V2.5 Unsure which of double-barrelled item to consider (e.g. get around inside/outside) | | |  |  |  |
|  |  | V2.6 Thinks about someone else for some domains but not others | | |  |  |  |
|  |  | V2.7 Current task being framed in some way by previous task (e.g. use of language from participant) | | |  |  |  |
|  |  |  |  |  |  |  |  |
| **Amendments (A)** |  | A2 Things they don't like or would like to have changed | | |  |  |  |
| **Feedback (F)** | F1 Survey easy / straightforward | F2.1 Perceive survey as easy, but respondent made errors | | | F3.1 Found confusing/difficult | F4.1 Strange question to ask |  |
|  |  | F2.2 Found survey repetitive/boring | | |  |  |  |
| **Use of the survey (U)** | U1.1 Varied/mixed sample |  |  |  |  |  |  |
|  | U1.2 Mixed methods - qualitative |  |  |  |  |  |  |
|  | U1.3 Good to ask the public |  |  |  |  |  |  |
|  | U1.4 Concern regarding speeders/motivation |  |  |  |  |  |  |
|  | U1.5 Difficult/confusing survey/ understanding |  |  |  |  |  |  |

## Table S4.2 Frequency of concerns identified and example quotes

| **Level** | **Issue Identified** | **Number of interviews raised** | ***Example quotes**** |
| --- | --- | --- | --- |
| **RANKING** | |  |  |
| Minor | R2a.1 Assumes change or intervention is possible (e.g. took more rest) to described state | 6 | *“And then things like anxiety, loneliness, they can all be sorted out, through counselling” (M5059-TP1) “Physical pain - can actually be dealt with using drugs or whatever” (M70+-TP5) "...feeling lonely, maybe, I feel that you can change by just doing something." (F4049_CM5) "I feel like it's more easy to manage sometimes my anxiety and depression is managed through medication" (F3039_CM8) "...I think that's something that you can fix with mindfulness and stuff like that and just thinking about stuff" (M3039_CM9) "...at least the other physical pain can be rectified" (F3039_CM10)* |
|  | R2a.2 Assumes interactions between domains | 12 | *“Physical pain would likely have an impact on somebody’s mental health as well. So, I feel that one would be at the top of the list” (M1830-TP9) "I didn't spot day-to-day activity been able to do that. I would probably drop that in just under the getting around. Because again … that would lead to lack of control. And that line stem from being in severe, physical pain " (M5059_CM0) “pain would be linked with doing day to day activities... Lack of control. Then again if I'm in pain I can't do that. The anxious I would be because I'm in pain” (M6069-CM1)  "Physical pain being very severe.... I think that would probably give you all the other issues if you had that." (F6069_CM2) "Most of all the time is something anxiety issues. you can't leave the house or affection behaviour." (M4049_CM3) "I think for me, physical pain, and getting and being able to go around would lead to mental anguish anyway" F4049_CM5) "if you're in physical pain, it does affect your mood, affects everything that you can do really." (F6069_CM6) "if it was very severe for pain, it probably stopped me doing almost anything else" (M3039_CM7) "I feel like those three are kind of tied together, the exhaustion and not being able to do day-to-day activities and getting around" (F3039_CM8) "...feeling anxious because I always think this goes hand in hand, with sad or depressed. It's inevitable to feel anxious when these things happen." (F3039_CM10) "I feel like that would probably then cause all of the following things, if you're in that much pain, you're not gonna be able to do your destiny activities and you probably are going to be depressed and just struggling with your life in general" (F1830_TP11) "lack of control over your everyday life. That's pretty general because some of the criteria here could cause that to happen, I guess" (M70+_TP3)* |
| Major | R3.1 Rank was based on levels currently experienced | 3 | *“none of these really apply” (F70+_TP6) “Getting around Unable, doing day to day unable, activities unable, feeling exhausted most or all of the time, but it doesn't really affect me because I get out and about” (F70+_TP7)  "It’s not very well laid out, really? Because it should be different options on there with sort of slight pain, medium pain. Then you can work around it a little bit better." (M6069_CM1)* |
|  | R3.2 Ranking in the reverse order | 2 | *"I probably think the depression is the worst one. Because it affects everything, you do all your thoughts and it's a kind of invisible and invisible illness. So, people don't really understand and you get comments like, oh you’re just a bit sad. So that'll be my number nine at the end." (M4049_CM4) "So I'm gonna start with maybe the least bad is that ok ... so, God, they're all really bad. And okay, I'm gonna go trouble concentrating as the least bad." (F1830_TP12)* |
| **SWING-WEIGHTING** | |  |  |
| Minor | S2a.1 Assumes interactions between domains | 9 | *"...because the way I see, is they are inter-linked” (M1830_TP10) "How many points would you give to this improvement? I would say if the first two improvements took place, then this would be reduced amount" (F70+_TP6)  "Getting around unable. ... Give me 80 because obviously that affects your day-to-day life" (M4049_CM3) "So considering improvement for feeling most or all time to feeling exhausted ... I know, this is one of them things that can improve as I improve the depression issues. So. I guess if I improve one, that'll have a positive effect on this as well" (M4049_CM4) "...physical pain, very severe ...would come with many other things like being depressed, feeling, exhausted" (M3039_CM7)  "I'm going to give this the highest score because it comes into mobility." (M3039_CM9)* ***"****And day-to-day activities I think that would link in probably with the getting around one.****"*** (M5059_CM0) "The things like that physical pain, unable to move. Obviously, would tend to increase your lack of control over your day-to-day life – you’d be relying on other people" (M70+_TP3) "And if wasn't able to get about that, that I would lead I would suggest probably to some mental deficiency as time went on" (M70+_TP8) |
|  | S2b.2 Struggled to complete task independently | 2 | *"Okay. I'm not sure what want me to do on that one...I’m not sure I understand what the point is here" (M6069_CM1)  "It's sort of the reverse of what I'm thinking it would be. So, I'd be thinking 100 is the worst thing and it's asking me, what score I would give something for it not to happen...so it’s a bit obtuse." (M70+_TP2)* |
|  | S2c.3 Difficulties with EQ-HWB descriptor e.g. no control/control double negative, control is vague | 2 | *"Lack of control of your day to day, life. I think I'll give that 60. It’s too vague." (M4049_CM3) "Because with this one I kind of have to rephrase it in my head, it's kind of an unnatural construction of language in a way if you sort of go consider an improvement from doing day to day activities unable to doing this day activities." (F1830_TP13)* |
|  | S2b.4 Answers most at 100 without clear reasoning | 2 | *"I'd want total control so that'd be a hundred wouldn't it? [...]"But I wouldn't want to feed sad or depressed so say 90" […] "Day to day activities, I'd want those so that would be hundred" […] "Being lonely, yeah I hat feeling lonely - a hundred" (F70+_TP4) "Trouble concentrating, a hundred, you need to be there. Feel exhausted none to all the time. Again, a hundred. Consider feeling lonely. And then that’s still important. 100." (F3039_CM11)* |
|  | S2a.5 Assumes intervention possible to described state | 4 | *"And the sad or depressed. Again, that's up to me - you can do things yourself to improve your mood” (M70+_TP8),  "I think being able to do day today activities, probably quite really important but probably not quite as much of a priority. Because I could definitely get help with that." (F6069_CM2) "I do have anxiety, but I can control it a little bit" (F6069_CM6) "...I know there's an easy fix to being exhausted. It's go to bed..." (M3039_CM9)* |
|  | S2b.6 Response based on how likely having no problems would be in a domain | 3 | *"I feel like obviously, there's always things in life that you can't control,... " (F3039_CM8) "I think loneliness is probably something that nearly everyone experiences at some stage is so a hundred improvements, probably not practical. So again, I'd probably say if you could improve it by our 70...I kind of thought about maybe somebody elderly. And who couldn't get around. [...]You can’t go from, maybe using a walking frame or using a wheelchair to suddenly being 25 again, in being able to run upstairs and kind of climb into loft and all things like that. And I think sometimes things are…that's not practical" (M5059_CM0) "Lack of control on your day-to-day life most of time, I think everyone would love control over everything, but it's not possible." (M5059_TP1)* |
|  | S2c.7 Response based on having no problems in a domain not being ideal | 3 | *"But I also think that anxiety is a useful emotion to have because sometimes it stops, you doing things, that might be really dangerous. So, I would say having it, none of the time might also be a problem...I think those perfectly normal human feelings. I think life would be a bit strange never feeling sad...So, having complete control over my entire life. Maybe wouldn't be that good." "...exhaustion that's actually also be useful..." (F6069_CM2) "it's a necessary evil sometimes exhaustion so I think sometimes I thrive off knowing that, I'm tired, because I've done a lot" (M3039_CM9) "It’s good to be exhausted sometimes." (F3039_CM12)* |
|  | S2b.9 Reference domain not referred to in rating | 8 | *And feeling exhausted, most of all of the time. That needs 100 points. I am exhausted all the time, and this one probably has the biggest impact on my life (F3039_CM8) "What number should okay, …I think considering to improve lack of control over day to day. Yeah, that's quite important [rates at 100]." (F3039_CM11) "That needs 100 points. I am exhausted all the time, and this one probably has the biggest impact on my life" (F3039_CM12)* |
|  | S2b.10 Confused initially but then interprets as expected | 4 | *"I mean it's difficult because sometimes there are some that are fairly equal to you but having to assign them a number, I started thinking slightly differently, I think." (F5059_EM1) "I was thinking that higher was more impact. It was worse. More was better, and I was. it'll be around looking at the first example. yeah, so none of the times a hundred which means that that's the best scenario. Yeah. whereas I was thinking it was the other way around." (M4049_CM3) "It's difficult, isn't it? Because I think all of these, I would want to be at the top. I think because that gives you perfection." (M3039_CM9) "it's sort of confused me for a second, why I couldn't move this slider about? [note refers to the first slider]" (M1830_TP10)* |
|  | S2b.11 Assumes can't have more than one domain rated at 100 | 4 | *"I think you're slightly restricted by the fact that the first rank that you put on the previous page is automatically given a hundred. And since you've already said, that's the most significant thing for you. You feel you can't really put anything else at the same level." (M70+_TP3)  "I probably won't give anything that high a score, but because that one was on a hundred. I felt like I got to put, which one was it? The day-to-day activities. Because I'd put them pretty much on a par. Felt like that one ought to be a hundred as well." (M5059_CM0) "This is the most important improvement. I would feel like I could only give one thing a hundred points and maybe I would have to try and even if lots of things were 99..." (M3039_CM7)*  *“Okay, so you can give multiple ones a score of 100. " (Funder30-TP11)* |
|  | S2b.12 Tries to score reference dimension | 3 | *"Okay. I'm not sure what want me to do on that one [participant tries to move the values for the reference which is fixed]" (M6069_CM1) "Physical pain from very severe to none. [tries to score reference dimension] Okay. Sorry" (F6069_CM2) "Yeah, I would go with hundred. [Tries to score the reference dimension]" (F3039_CM12)* |
|  | S2b.13 Changes response in response to discussion | 1 | *"I suppose you think about it, maybe go back to it but yes, maybe I'm so I was rushing a little bit there. I shouldn't do but and yeah, I mean it is important to sort of yeah." (F6069_CM6)* |
| Major | S3.1 Response based on levels currently experienced | 2 | *"Consider an improvement getting around. So again, there is a little bit of this just in the fact that I can't go hiking at the moment. But in general, I can get around, fine, I can drive, I can walk. I just can't do the long hikes that I used to do" (M4049_CM4) "That's wrong. I don't have a problem going around at all" (F70+_TP6)* |
|  | S3.2 Scoring as 100 because the domain would be completely improved i.e. 100% or because you would choose to be at 100 for each | 4 | *“you would want a hundred percent on all of them” (F50-59_EM1)  “How many points would you give this improvement? Obviously a hundred […] it would be 100% improvement... "how can you not give a hundred percent? I don't understand where you are coming from in this. If it was there, none of the time." (F70+_TP6) "it would be completely irrational to not want to fix everything and get everything better” (M1830_TP10) "… and I wouldn’t want that. Feeling lonely none of the time well it's not nice to be lonely [...] That's not good either" [note scoring most at 100] (F70+_TP7)* |
|  | S3.3 Lack of understanding about 0 to 100 scale | 2 | *“I haven't got a clue what that means” (M70+-TP5);  "Yeah, you got so many points to give in it. Where do you want to focus your efforts? I suppose in that’s what you're saying with this exercise here, where would you focus your efforts? what's your priorities? How much effort would you put in to get in control of that? And then I'm thinking more in percentage points rather than actual individual points. So, I spent 50% of time trying to sort out my concentration levels, it's resolvable, but do you have to put all your effort into it, probably not." (M5059_TP1) [note here the points allocated is a combination of importance and how 'resolvable' the problem is perceived to be]* |
| **LEVELS-RATING TASK** | |  |  |
| Minor | L2c.1 EQ-HWB: Level descriptors are not distinguishable. | 4 | *"only occasionally is in a sense quite a vague term. In a sense, it might be easier if it's said, you're feeling lonely only 75% of the time or only 25% of the time" (M70+_TP3) "Occasionally, that's still pretty good. So, you'd feel occasionally anxious I'd say. That's what for most people would be what classes are normal state? And the difference between occasionally and sometimes? So, I wouldn’t really know what the difference between occasionally and sometimes is. The two ways that I would say, are almost the same" (M5059_CM0) "And it's kind of ambiguous, because it depends on what kind of difficulty it is." (M4049_CM3)  "And okay, so that's 90. And even that would be next one would be 90." [note only occasionally and sometimes rated the same] (F70+_TP4)* |
|  | L2b.2 Confused the levels of the slider with judgement of frequency | 1 | *sorry I find it so hard to sort of think about that on a scale of zero to 100 because it's like do you mean if I say I'm feeling lonely sometimes am I actually feeling lonely like 50% of the time (F1830_TP13)* |
|  | L2b.3 Struggled to complete task independently, expressed confusion | 7 | *"I find it strange. I'll be honest I don't completely understand it and I'm certainly not thick" (F70+-TP4) "Which again is a little bit confusing. I can’t see the point in it myself but that's just me" (F70+-TP5) "Well this is quite hard you know, I'm not thick by any means, but this is quite hard" (F70+-TP6)  "The confusing bit is getting around some difficult and getting around a lot of difficulty" (M6069_CM1)  "No difficulty as a value of 100. But then, it feels backwards, it doesn't feel like the bar is the right way, it feels like how many things? How many things are slightly difficult? A lot of things are slightly difficult, so it feels like the point it should be at the top of the bar" (M4049_CM4)  "Yeah, I can't really get my head around. It's alright." (F6069_CM6) "But then here, if you look at the reference, none of the time is hundred so I don't want it to be at any time. So, shouldn't hundred be the one for [highlights only occasionally]" (F3039_CM12)* |
|  | L2b.4 Confused by order but self-corrects or after pop-up box | 4 | *Okay, so it's rating physical pain between naught and hundred basically and the severity. So severe, it's gonna be right up there*. [note – puts 4th category at nearly 100]*: no, it's the other end isn't (M70+_TP2) "I think it might be that wording of where it says ‘no difficulty’ has a value of a hundred because I think of nothing like no difficulty as a zero. So that might be what confused me about it." (F6069_CM2) "so none of the times a hundred which means that that's the best scenario. Yeah. whereas I was thinking it was the other way around" (M4049_CM3) "Yeah. I mean if you weren't here this would have caused me to go back and […] look again. So, it's kind of doing what it’s supposed to do." (M4049_CM4)* |
|  | L2b.5 Uses the ends of the scale to highlight good/bad providing an ordinal preference | 3 | *Scoring most at zero because of dislike of the state description "No, I wouldn't like that" (F+70-TP7)  "even slight difficulty's bad for me […] it's got to go back to five for that (F+70-TP4)  "Getting around. Some difficulty. [rates 100,90,0] Sometimes [Lonely rates 100,100,0]. trouble concentrating. Some occasionally, 100. Sometimes I’ll give it a hundred often I’ll give it Zero." (F3039_CM11)* |
|  | L2b.6 Tries to move fixed top or bottom | 11 | *Yeah. it won’t let me move that one interesting (M5059-TP1) " And back up there. When I go there and … [tries to change the bottom]" (M6069_CM1) "Okay. All…So that's zero, So I can't change that." (F4049_CM5) "So that will be zero, wouldn't it? don't know. [tries to move the fixed top one] It's not gonna let me do it." (F6069_CM6) "No, I was trying, I didn't realize it’s already marked the bottom to where needed to be." (F3039_CM10) "Most of the time that’s the same though, It's really as well. Maybe. [tries to rate worst level]" (F3039_CM11)* |
|  | L2b.7 Reference bias i.e. rates levels based on top or bottom levels, or absolute vs relative | 4 | *"the one before I was like, that was so easy. But I just obviously completely misunderstood that one ...Yeah, certainly made more sense when you explained it that way" (F1830_TP12)  "it is really interesting seeing how differently your brain works through a problem just looking at it the other way around." (F1830_TP11)  "Yeah, I'd say it's just a function of I didn't really think about where the zero was being unable, but I think, again a lot of difficulty, I wouldn't want to be near it. But that still means something can be done. Ultimately compared to unable means nothing can be done. So, with that pointed out to me, I would probably move this more that way. But again, it's just like I sort of missed the nuance between unable and still technically possible. (M1830_TP10) "But then, 70 or so. So sometimes. So, I think 75. Okay, then often is not very good at all. I think there’s a big gap between sometimes and often so, … I'm gonna say, I’m gonna say 25." (M4049_CM3)* |
|  | L2a.9 Assumes intervention | 1 | *"I don't mind having mild pain because I can live with it. I can treat it and you can medicate for it. […] I can fix that easily by having a good sleep." (M3039_CM9)* |
|  | L2b.10 Changes their answer when given interpretation | 2 | *"And maybe that is a little bit high and Only yeah, I suppose it's maybe put it down a bit, just maybe that " (F6069_CM6)* |
| Major | L3.1 Response based on levels currently experienced | 3 | *"I very rarely feel lonely" (M70+_TP5) "This is, so I have good and bad days. My scores from a good day to a bad day will be completely different, completely opposite in the scales" (M6069_CM1)  "I'm trying to get my head around. How these are working? […] So, if I'm reading this right, I would have all of my daily activities basically, have some difficulty. Some of them have quite a lot have some difficulty. At the moment. Probably about half of them have a lot of difficulty. " (M4049_CM4)* |
|  | L3.2 Interprets the 0-100 the wrong way round or confused by the 0 to 100 scale | 5 | *"Since I can't change that [referring to the bottom response] there’s nothing I can do really so" (M5059-TP1) [note this is in response to the pop-up box alerting to the error] "That's slight difficult it. Nice. Because that means 10%. I might have difficulty doing summat. Yeah, no more. I think. [sets all the levels at 90 and hits continue] " (M6069_CM1) "Not thinking clearly only occasionally, Right. So Maybe. And we'll go down a bit further. so sometimes yeah, that yeah. You go, that's and up to that. [sets at 19, 31, 76]" (F6069_CM6) "And yeah, I think it's hard for me to do, I think for me a lot of this is all or nothing and so then it's hard to then band things in a percentage because I think realistically, no one wants to aim for having slight difficulties. So it's difficult to then say, how would I prioritize that in terms of perfection. I think I'd rather be 100 or 0 if that makes sense" (M3039_CM9) " So I think…will be the Severe pain. Definitely 95 is only one step down from very severe" F3039_CM10)* |
|  | L3.2a Interprets the 0-100 the wrong way round or confused by the 0 to 100 scale AND ignores the pop-up error message | 4 | *"I think I'm happy with them. Yeah. [participant continues despite error message]" (F3039_CM11) "I would still go with the hundred [for all the levels] because it's one of the basic things" F3039_CM12)* |
|  | L3.3 Scores as 100 because the domain would be 100% improved | 1 | *What would you feel like to experience […] I would say they would all feel 100%. To. How can you say just because only occasionally it would be less than that. Because it wouldn’t - it would all be 100%. (F70+-TP6)* |
|  | L3.4 Confused (based on verbal or data) after previously getting it correct | 6 | *Yeah, sorry. Yeah, you're right. I've done that. Completely wrong way around. It's 25. Yeah. (M5059_CM0) "Physical pain. This looks like. The numbers hundred severe. So, this looks like the same as before, but it's just been reversed. Or is it?" (M4049_CM3) "Yeah, I've got that the wrong way. Sorry, I'm not right. So, everything has some difficulty. Yeah, so I'm gonna be that way. So yeah. No difficult. No difficulty has a value of a hundred? Yes, that's right. so, I'm finding even the small things slightly difficult. The big things" (M4049_CM4)* |
|  |  |  |  |
| **PAIRWISE DEAD TASK** | |  |  |
| Minor | C2a.1 Assumes question is about euthanasia | 5 | *"This is obviously quite an important question we have right now. I mean, it's already making me think of all the cases of euthanasia and all the discussion in the media about that." (M1830-TP9)  "It's a very stark one this. Ummm, this is something it's a bit almost like a moral question and I struggle with kind of things like assisted dying and things like […] I've kind of don't know. I have no strong opinion because it's not something I've ever been in that situation. But I think it's such a minefield and… difficult. Thing that I don't almost think there's anything worse than being dead." (M5059_CM0) "And so I think that kind of euthanasia argument" (F6069-CM2)  "... I think where people's quality of life is being so severely and encroached upon by these kind of things. Then it's a much better option to at least allow them, if they're in a reasonable state of mind and legally get help in assisted dying" (M3039_CM7) "I think the first thing that springs to mind is the likes of euthanasia. and whether it's potentially morally acceptable for someone who meets a criteria where they don't have a good quality of life or any quality of life to make a decision as to die." (M3039_CM9)* |
|  | C2a.2 Assumes change is possible (e.g. intervention) | 4 | *"I would say that even if I was in severe pain. I would still have hope that my condition would get better" (M4049_CM3)  "...anxiety is a short-term episode" (M4049_CM4) "You can probably get the help and just overcome them all." (F3039_CM11) "everything can be managed for pain, feeling sad. I think there are a lot of resources for anybody in this time and age" (F3039_CM12)* |
| **ANCHORING DEAD TASK** | |  |  |
| Minor | D2a.2 Assumes question is about euthanasia | 1 | *"Shall we say it’s a personal decision that people have to make. And certainly, I’ve thought about at what Quantity of my life would I consider going to Switzerland or The Netherlands. Or even to Canada. To go to one of the specialized clinics. Because there's nothing available in this country and it is really a personal decision, what people say it's going to be correct for them and there's nothing for me to say" (M70+-TP5)* |
| Minor | D2b.1 Number given represents ordinal preference only | 1 | *"But partly, it's a cop out because I can't really decide. I can't really put a number on the benefits or how much better I would feel in relation to being dead compared with all those physical and mental problems" (M70+-TP3)* |
|  | D2b.3 Confused initially but then interprets as expected | 2 | *Again, it's A real struggle. Understanding what they are getting out here. It's the top of the scale is no health or being problems. The bottom of the scale is every problem. (M70+_TP2)* |
|  | D2b.4 Dead at zero because dead has no quality of life | 1 | *"I find it really tricky because the scale is talking about your health and wellbeing, So, I mean if you're dead you haven't got any health or wellbeing. I mean, … I'd almost feel like I'd want to put scenario on a minus of the scale" (F6069_CM2)* |
|  | D2b.5 Substantially changed following discussion | 1 | *[TP asks, "How did you come up with 20 years as a figure to choose?"] "I'm not sure I know definitely below 50, I don't know. I think 10 is just maybe too close to A because if I'm saying being dead it's better than A But then, that is kind of what I think, it's only slightly better. It's not way better. I thought maybe I actually feel better putting another 10 because they're both really not good" [Switches response to 10] (F1830_TP12)* |
| Major | D3.1 Scores at 100 because it is the opposite to the worse state or 100% better than worse state | 5 | *“So you're saying scenario A is leading to zero compared to no health or wellbeing problems. Therefore, the exact opposite being dead is therefore the exact opposite. At a hundred...Obviously, being dead. So it's gonna have to be the exact opposite, isn't it?” (M5059-TP1); "Hundred percent, If I can't get around. So, sat in the chair by doing nothing. One of my big nightmares. the day today activities, I have people to do stuff for me all day Get me through wash me and all that lot. It's another thing I don't want to happen...." (M6069_CM1); "No health and wellbeing problems has a value of 100 being dead has value 0. [100] ..." F3039_CM11); "I would say Scenario B is 100% better than Scenario A" (F70+_TP6); "having no health problems or being dead – there’s no choice is there" (F70+_TP7)* |
|  | D3.2 Unable to complete task | 3 | *"I'm utterly confused by it, basically. This is confused me here." (M5059_TP1); "I honestly would not really have a clue. The choice of. There is no good way of doing it." (M70+_TP5); "This is very difficult to follow...because it would have to be significant improvements in everything in Scenario A to make Scenario B less than 100% better" [Unable to respond other than as 100% even with support] (F70+_TP6)* |
|  | D3.3 Reinterprets the VAS to rate where they would want to be | 3 | *"I wouldn't want to be dead so it’s at the top isn’t it - I've gone to the wrong level. Yes, no health or wellbeing problems is at the top so you can take that back up to 100." (F70+_TP4);  "Yeah, because I didn't want any health problems. I didn't want to be dead" (F70+_TP7); [Places dead at 100] "well it says, if I have no health or wellbeing problems, as opposed to being dead, is there a choice?" (M70+_TP8)* |
|  | D3.4 Reinterprets the VAS as own health | 1 | *"And I guess I guess it's a personal thing on your perspective of your life at the moment". (M4049_CM4)* |
|  | D3.5 Reinterprets the top of the scale in relation to dead i.e. when dead you have no health and wellbeing problems | 3 | *"...perversely I suppose it's at a hundred really because at the point where you're dead you don't have any of those problems anymore" (M3039_CM7); "Because it says, so for me in my head being dead means no health wellbeing problems. But within the question, I don't know if that's what it's trying to get at." (F30-39_CM10)  "It has to be a hundred. You would have no health problems. You'd have no well-being problems." (F5059_EM1)* |
|  | D3.6 Misunderstanding the task e.g. thinking it was about personality | 1 | *"I mean, if people are putting this 10 or 20, I feel like they might be quite negative people, they may have given up a little bit. And obviously if they've put it higher than me, then they're probably more positive, more focused and more motivated than me." (M4049-CM4)* |
|  | D3.7 Reinterprets scale to mean proportion time alive | 1 | *"People are very persistent and at least in my opinion, people are persistent, and people want to survive. [...] So, they would at least experience and then once, they believe that, this is not working out for me and the only then any person I think I feel would want to be gone." (F6069_CM12);* |

* *Where relevant a quote is extracted from each interview identified as having that concern, however, this is not the case where concern is based on interviewer observation and data input*

## Table S4.3 Example quotes for other themes

| Code | Selected quotes |
| --- | --- |
| Confusion with scales | “*I just found it - the wording very confusing - well the scales, the changing of scales from naught to 100 and back to 100 and so on it probably makes sense from a statistical point of view. But does it make sense from an end user point of view?”* (M70+_TP2)  “*I just think that's the way most of our, we're geared, isn't it? That if you have a higher number, it's worse than if you have lower number. And if you ask me my health, I put a hundred. So, on that original one, what's your health today? And I put a hundred because it was good. And where's this is the opposite way around. No, it's not. It's none of the time you are feeling good. It's bizarre. Okay. It's my head's going. But I'm thinking of it seems weird.*.” (F5059_EM1)  "*I think it might be that wording of where it says ‘no difficulty’ has a value of a hundred because I think of nothing like no difficulty as a zero. So that might be what confused me about it*." (F6069_CM2)  *“So, I think a better way to make this survey less a mistake where they would be to swap it around. So, physical very serious should be a hundred … because none in our heads is zero.”* (F3039_CM10) |
| Insufficient information provided in task | “*… it doesn't give me more information. It doesn't say for how long, doesn't say your mental faculties are affected*.” (M4049_CM3) |
| Issues valuing EQ-HWB | “*using the terms ‘occasionally’ or ‘sometimes’ that sort of thing, I think having some sort of scale that you could use to be more specific, would have made it easier because I was sort of chasing around, trying to decide -What does occasionally mean.*” (M70+_TP3) |
| Feedback on tasks: found easy (F1) | “*all very easy, straightforward*” (M4049_CM3) |
| Feedback on tasks: found difficult (F3.1) | *“I think you have to have above average intelligence to even comprehend what you are being ask of on occasion*” (M70+_TP8)  “*It was a bit hard to grasp what you were sort of looking for really - with the rating system, I found that quite difficult really*.” (F70+_TP6) |
| Recommended requiring reading additional details | “*I think it just felt a bit like it is optional to read some of the information that I think that information was quite useful…and if I'd been doing it without you, there potentially might have skipped that information. And I think I might have answered differently if I didn't have it*. (F6069_CM2)  “*I think this explains it here, but you think it'd be less important hidden down in a show details button. But this is the most important part of this is explaining sort of what you explained*.” (M1830_TP10) |
| Recommended including why survey is being done | “*It would be really good if there was a summary of why they were doing it at the end so people could understand. Getting people to do things is one thing but helping them to understand why they're doing it and what effect they will have can be a really good motivator*.” (M4049_CM4) |
| Recommended changing reversing the order of levels-rating task | “*I would have physical pain none at the bottom. And severe physical pain at the top… Just reversing your thing to make it look sensible.*” (M70+_TP5) |

## Table S4.4 Participant details

|  | Frequency (n=27) |
| --- | --- |
| Age group: |  |
| Under30 | 5 |
| 30 to 39 | 6 |
| 40 to 49 | 3 |
| 50 to 59 | 3 |
| 60 to 69 | 3 |
| 70+ | 7 |
| Gender: Female | 14 |
| Education: |  |
| No education beyond minimal school leaving age | 2 |
| Post school education (not a degree) | 9 |
| Degree or equivalent | 16 |
| Self-report a long-standing illness of which: | 14 |
| Physical health problem | 6 |
| Mental health problem | 5 |
| Combined physical and mental health problems | 3 |
| Informal caregiver for “someone sick, disabled or elderly whom you look after or give special help to" | 4 |
| Experienced serious illness or disability in yourself or others (e.g. family members, close friends) | 21 |

# 5. S5 Reflection on researcher biases

All researchers have been involved in previous research projects collecting data using the OPUF method and other valuation methods. Some were also involved in the development of OPUF and the development of the EQ-HWB-S. The research team held a general belief that both the OPUF method and the EQ-HWB-S are valuable, or at least promising, approaches. None of the research participants were known to the interviewers, nor were they aware of any involvement of the researchers in the development of the OPUF.
